# Editorial: Animal Welfare Assessment: Edition 2

**DOI:** 10.3389/fvets.2021.736827

**Published:** 2021-08-11

**Authors:** Edward Narayan, Alan G. McElligott

**Affiliations:** ^1^School of Agriculture and Food Sciences, Faculty of Science, The University of Queensland, St Lucia, QLD, Australia; ^2^Queensland Alliance for Agriculture and Food Innovation, The University of Queensland, St Lucia, QLD, Australia; ^3^Jockey Club College of Veterinary Medicine and Life Sciences, City University of Hong Kong, Kowloon, China

**Keywords:** precision livestock farming, health, animal welfare, physiology and behavior, human-animal interaction

Animal welfare is an important dimension of human-animal interaction in managed settings such as farms and zoos. This field of research can also be a powerful driver to continuously improve the traditional animal production systems to ensure that the animals are able to meet basic requirements of five freedoms (freedom from pain, injury and disease, freedom from fear and distress, freedom from discomfort, freedom to express normal behavior and freedom from hunger and thirst). The recently developed five domains model is also internationally recognized and it attempts to provide an understanding of the emotions of animals (affective state) in response to human interventions. Animal welfare legislation is a complex topic; however, consumer awareness associated with the methods of animal production, health and biosecurity risks increasingly demand stronger investment into research and innovation to continually improve animal welfare standards.

In Edition 2 of the Animal Welfare Assessment *Topic*, we showcased a collection of 13 peer reviewed articles which highlight advancements in animal welfare assessment methods across animal production systems. It includes works of animal welfare experts, veterinarians, animal physiologists and animal managers that will generate a healthy discussion and showcase latest studies working toward finding the harmony between animal performance, health and welfare.

Navarro et al. presented a pharmacological intervention to improving piglet immunity using oral Meloxicam administration to multiparous sows. Early neonatal care of piglets is vital to their survival. The researchers were able to demonstrate that administration of meloxicam orally at the beginning of farrowing in multiparous sows increased immunoglobin and cytokine concentrations in colostrum, improving both humoral and cellular immune response of piglets.

Rodger et al. further studied an app called the health-related quality of life (HRQL) instrument (VetMetrica™) that generates scores in four domains of quality of life in dogs—Energetic and Enthusiastic (E/E), Happy and Content (H/C), Active and Comfortable (A/C), and Calm and Relaxed (C/R). Importantly, the app was able to pick up the disagreement between owner opinion in health status and clinical evidence of chronic disease (40% disagreement), however scores of HRQL were higher in healthy dogs with no clinical information.

Chronic stress can be a significant problem in intensive animal production systems, hence robust quantitative tools are required to measure and evaluate the potential of chronic stress. In their paper, Wiechers et al. studied chronic stress between two different farrowing systems in pigs. Researchers used hair to determine cortisol levels of sows managed either in farrowing crates or in a loose-housing system. They did not find any significant difference in hair cortisol concentrations between the two treatments, however the researchers pointed caution in the potential variation of results due to site of sampling as well as potential modulation of the HPA-axis under exposure to long-term stress.

Hematological methods or blood testing can also boost animal welfare assessment. Seibel et al. discussed the technical developments and opportunities for fish health and welfare monitoring in aquaculture programs. In another study, Ramos et al. discussed important aspects of stress and welfare in fish, highlighting the need for further research based on stress assessment in early life-history stages of fish including focus on egg transport and larval handling.

Emerging animal industries are gaining popularity around the world, such as camel farming. Padalino and Menchetti studied the welfare of camels by applying the principles of Five Freedoms using the Welfare Quality® and AWIN methods adapted to camels. The researchers provided three levels of assessment including (i) Caretaker, (ii) Herd, and (iii) Animal and provided recording sheet for use by Camel producers.

Precision livestock farming (PLF) technologies are gaining popularity as a digitized sensor-based tool to improve the welfare assessment of farm animals. Stygar et al. applied the PRISMA guidelines to evaluate validated and commercially available PLF technologies for welfare assessment of dairy cattle. The study suggests that sensor-based technologies such as accelerometers, milk quality and feeding sensors are useful for assessing welfare status. However, currently available PLF technologies needs to be improved with external validation to boost the assessment of cattle behavior (including calves and heifers) in a reliable way.

In their study, Gómez et al. conducted a literature review on the capability of PLF technologies to contribute to the assessment of pig welfare. Researchers identified 83 PLF technologies commercially available for pigs. However, only 5% were externally validated using a different population than used for system building. Researchers highlighted the need for further validation studies to improve robustness of available technologies as appropriate pig welfare indicators.

Tuyttens et al. discussed the improvements and application specifications of the Welfare Quality® protocol as a user-friendly tool for cost- and time-efficient on-farm monitoring of dairy cattle welfare through application of discrete and continuous animal-based measures feeding into a welfare index (WI). The researchers highlighted that the WI captures most of the welfare key issues dairy cattle, however a list of parameters need to be included as a point of reference to ensure that the data is interpreted correctly using the available anima-based measures.

Brscic et al. evaluated the use of animal-based measures (ABM) in farm animal welfare assessment to standardize terminology that could be applied across sectors. They found that the term ABM was not standardized across sectors and was hardly a common language for different stakeholders. IN order to harmonize the use of ABM in the scientific literature, it was suggested that commonly accepted abbreviations of ABM should be made available in scientific journals.

Lee and Campbell studied virtual fencing technology in cattle to further evaluate the suitability of aversive method such as electrical stimulus. The researchers suggested further research to understand physiological and behavioral responses of animals to see how the virtual fencing technology can be functional animal welfare tool.

In another study, Perea et al. studied the influence of littermate and sex on hormonal and behavioral data from carolic restricted (CR) group housed mice. They showed that grouped male littermates and grouped female male showed less aggressive behavior and physiological stress (measured using serum ACTH levels) during CR, highlighting the welfare benefits of grouping related mice during implementation of CR.

Hempstead et al. studied the welfare assessment of dairy goat farms in the midwestern US, with focus on lactating dairy goats to identify potential welfare issues. Using principal components analysis, the researchers were able to identify physical indicators of welfare issues that will be valuable information to improve goat welfare in dairy industry.

Collectively, the *Topic* further highlights the latest innovations that are helping to boost animal welfare assessment across industries.

## Author Contributions

EN conceptualized this special issue and collaborated with AM for the editorial role. Both authors contributed to the article and approved the submitted version.

## Conflict of Interest

The authors declare that the research was conducted in the absence of any commercial or financial relationships that could be construed as a potential conflict of interest.

## Publisher's Note

All claims expressed in this article are solely those of the authors and do not necessarily represent those of their affiliated organizations, or those of the publisher, the editors and the reviewers. Any product that may be evaluated in this article, or claim that may be made by its manufacturer, is not guaranteed or endorsed by the publisher.

